# Correction: Profiling the impact of the promoters on CRISPR-Cas12a system in human cells

**DOI:** 10.1186/s11658-023-00502-4

**Published:** 2023-10-19

**Authors:** Jinhe Li, Qingchun Liang, HuaPing Zhou, Ming Zhou, Hongxin Huang

**Affiliations:** 1 Affiliated Cancer Hospital & Institute of Guangzhou Medical University, Guangzhou, 510095 China; 2 The Third Affiliated Hospital of Southern Medical University, Guangzhou, 510630 China; 3 Guangzhou Key Laboratory of Neuropathic Pain Mechanism at Spinal Cord Level, Guangzhou, 510630 China

**Correction: Cellular & Molecular Biology Letters (2023) 28:41** 10.1186/s11658-023-00454-9

Following publication of the original article [1], we have been informed that the name of author Qingchun Liang has a spelling.

The incorrect name is: Qinchun Liang

The correct name is: Qingchun Liang
