# Supplementary material for: Profiling the impact of the promoters on CRISPR-Cas12a system in human cells
Source: Cell Mol Biol Lett. 2023 May 17;28:41. doi: 10.1186/s11658-023-00454-9 (PMC10190037; doi:10.1186/s11658-023-00454-9)
Supplement: Supplementary file 1 — Additional file 1: DNA sequences and Tables. [file 11658_2023_454_MOESM1_ESM.pdf]

Additional file 1:

DNA sequences

- The CAG promoter (1719 bp)：

gacattgattattgactagttattaatagtaatcaattacggggtcattagttcatagcccatatatggagttccgcgttacataacttacggtaaatggcccgcctgg
ctgaccgcccaacgacccccgcccattgacgtcaataatgacgtatgttcccatagtaacgccaatagggactttccattgacgtcaatgggtggactatttacg
gtaaactgcccacttggcagtacatcaagtgtatcatatgccaagtacgccccctattgacgtcaatgacggtaaatggcccgcctggcattatgcccagtacat
gaccttatgggactttcctacttggcagtacatctacgtattagtcatcgctattaccatgggtcgaggtgagccccacgttctgcttcactctccccatctcccccc
ctccccacccccaattttgtatttatttattttttaattattttgtgcagcgatgggggcggggggggggggggcgcgcgccaggcggggcggggcggggcgag
gggcggggcggggcgaggcggagaggtgcggcggcagccaatcagagcggcgcgctccgaaagtttccttttatggcgaggcggcggcggcggcggc
cctataaaaagcgaagcgcgcggcgggcgggagtcgctgcgttgccttcgccccgtgccccgctccgcgccgcctcgcgccgcccgccccggctctgactg
accgcgttactcccacaggtgagcgggcgggacggcccttctcctccgggctgtaattagcgcttggtttaatgacggctcgtttcttttctgtggctgcgtgaaag
ccttaaagggctccgggagggccctttgtgcggggggagcggctcggggggtgcgtgcgtgtgtgtgtgcgtggggagcgccgcgtgcggcccgcgctgc
ccggcggctgtgagcgctgcgggcgcggcgcggggctttgtgcgctccgcgtgtgcgcgaggggagcgcggccgggggcggtgccccgcggtgcgggg
gggctgcgaggggaacaaaggctgcgtgcggggtgtgtgcgtgggggggtgagcagggggtgtgggcgcggcggtcgggctgtaacccccccctgcacc
cccctccccgagttgctgagcacggcccggcttcgggtgcggggctccgtacggggcgtggcgcggggctcgccgtgccgggcggggggtggcggcagg
tgggggtgccgggcggggcggggccgcctcgggccggggagggctcgggggaggggcgcggcggcccccggagcgccggcggctgtcgaggcgcg
gcgagccgcagccattgccttttatggtaatcgtgcgagagggcgcagggacttcctttgtcccaaatctgtgcggagccgaaatctgggaggcgccgccgca
ccccctctagcgggcgcggggcgaagcggtgcggcgccggcaggaaggaaatgggcggggagggccttcgtgcgtcgccgcgccgccgtccccttctcc
atctccagcctcggggctgtccgcaggggggacggctgccttcgggggggacggggcagggcggggttcggcttctggcgtgtgaccggcggctctagtgcct
ctgctaaccatgttcatgccttcttctttttcctacagctcctgggcaacgtgctggttattgtgctgtctcatcattttggcaaa

- The EF1a core promoter (212 bp)：

gggcagagcgcacatcgcccacagtccccgagaagttggggggaggggtcggcaattgatccggtgcctagagaaggtggcgcggggtaaactgggaa
agtgatgtcgtgtactggctccgcctttttcccgagggtgggggagaaccgtatataagtgcagtagtcgccgtgaacgttctttttcgcaacgggtttgccgccag
aacacag

- The CMV promoter (508 bp)：

cgttacataacttacggtaaatggcccgcctggctgaccgcccaacgacccccgcccattgacgtcaataatgacgtatgttcccatagtaacgccaataggg
actttccattgacgtcaatgggtggagtatttacggtaaactgcccacttggcagtacatcaagtgtatcatatgccaagtacgccccctattgacgtcaatgacg
gtaaatggcccgcctggcattatgcccagtacatgaccttatgggactttcctacttggcagtacatctacgtattagtcatcgctattaccatggtgatgcggttttg
gcagtacatcaatgggcgtggatagcggtttgactcacggggatttccaagtctccaccccattgacgtcaatgggagtttgttttggcaccaaaatcaacggga
ctttccaaaatgtcgtaacaactccgccccattgacgcaaatgggcggtaggcgtgtacggtgggaggtctatataagcagagct

- The PGK promoter (500 bp)：

gggtaggggaggcgcttttcccaaggcagtctggagcatgcgctttagcagccccgctgggcacttggcgctacacaagtggcctctggcctcgcacacattc
cacatccaccggtaggcgccaaccggctccgttctttggtggccccttcgcgccaccttctactcctcccctagtcaggaagttcccccccgccccgcagctcgc
gtcgtgcaggacgtgacaaatggaagtagcacgtctcactagtctcgtgcagatggacagcaccgctgagcaatggaagcgggtaggcctttggggcagc
ggccaatagcagctttgctccttcgctttctgggctcagAGGCTGGGAAGGGGTGGGTCCGGGGGCGGGCTCAGGGGCGGGC

TCAGgggcggggcgggcgcccgaaggtcctccggaggcccggcattctgcacgcttcaaaagcgcacgtctgccgcgctgttctcctcttcctcatctccg
ggcctttcg

- Expression of crRNA array

Red: U6 promoter, orange: AsCpf1-crRNA scaffold, crRNA targeted to DNMT1, EMX1, CTLA4, CCR5, SIRPa, RUNX1

AAGGTCGGGCAGGAAGAGGGCCTATTTCCCATGATTCCTTCATATTTGCATATACGATACAAGGCTGTTAGAG
AGATAATTAGAATTAATTTGACTGTAAACACAAAGATATTAGTACAAAATACGTGACGTAGAAAGTAATAATTTCT
TGGGTAGTTTGCAGTTTTAAAATTATGTTTTAAAATGGACTATCATATGCTTACCGTAACTTGAAAGTATTTCGAT
TTCTTGGCTTTATATATCTTGTGGAAAGGACGAAACACCGTAATTTCTACTCTTGTAGATGCTCAGCAGGCACC
TGCCTCAGCAATTTCTACTCTTGTAGATTCCTCCGGTTCTGGAACCACACCAATTTCTACTCTTGTAGATTATG
AAGTAGGTACTCAAAAGATAATTTCTACTCTTGTAGATTGGGCAACATGCTGGTCATCCTCAATTTCTACTCTT
GTAGATTGTCCGGGACACGTTTCCATTCTAATTTCTACTCTTGTAGATTGTCTCTCTGCCCCCTCCCCACTTTT
TTTTG

- The amplicon sequence for *DYRK1A*

TCAGCACAGAGCCATAGTCACTTGTGTTCAGAAACACTGACCTTATTTGATACACGTAAGGCTGAGTTTTATTT
AACAGATCTTTTAAGAAAACGTTATGATTCTTTGGTCCACCCCAAAAAGGTAGAAACAAATAATGGATCTTTAA
AAGGGGCAAGCTTCAGATTGTTTAGATTTTGTTTGTGAAATACTTCTCATTCCTCATTATTAAAATATAAAATGA
GTTTCTATTAATTTTTCTTAGTTTCTTTATAGATACTGATATAATTCATAATAATACCATTCTTATCTTAAAACCTTGT
CACACACAATGAAACTTTGCTGTTCACTGTCAGTTATAACTTACATGAGGTGACCCATTTCCATTCAAGGGT**TT
TA**GAAGCACATCAAGGACATTCTAAGGATGATTGACTTACACAATGATCTCTGAACATGCCTCCTGCCTTCTC
CTCACTCTT

- The amplicon sequence for *FANCF*

ACTGATTGGAACATCCGCGAAATGATACGCCTCTCTGCAATGCTATTGGTCGAAATGCATGTCAATCTCCCAG
CGTC**TTTA**TCCGTGTTCCTTGACTCTGGGCAACTTAAAAGCCCTAATACTTTTACTTTCGCCACACAAAGAGG
TTCTTCTTAGTGGAGGGAGAGCAGATGTAGGGCATCCTACCGAGAATTTCCGGAACCACGTGCGAGATGATG
CCAGTCAT

Table S1. The crRNAs and primers used in this study

Name	Sequence(5'-3')	location (hg19)
CCR5-1	TTTNTGGGCAACATGCTGGTCATCCTC	chr3 + 46414526 46414552
CCR5-2	TTTAAAGCAAACACAGCATGGACGACA	chr3 - 46414780 46414806
CCR5-3	TTTNGGATTCCCGAGTAGCAGATGACC	chr3 - 46415023 46415049
CD47-3	TTTNTCTCCCCTGCTTCCCACAACTAA	chr3 - 107805249 107805275
CD47-4	TTTNAAAACAGAAAGAACATACTCATA	chr3 + 107798052 107798078
SIRPa-S2	TTTNCTACAGTTACAACATTCCAACTG	chr20 + 1902671 1902697
SIRPa-S3	TTTNTGTCCGGGACACGTTTCCATTCT	chr20 - 1903070 1903096
CXCR4-S1	TTTNAAAGTCACATCTTGGCTAACTCC	chr2 + 136873755 136873781
CXCR4-S2	TTTNCCACGGCATCAACTGCCCAGAAG	chr2 + 136873196 136873222
PD1-S1	TTTNCCCAGAATCTCCAGTTCTGAGTC	chr2 + 242799735 242799761
PD1-S2	TTTNAGGCCCAGCCAGCACTCTGGCCT	chr2 - 242792483 242792509
CTLA4-1	TTTNTATGAAGTAGGTACTCAAAAGAT	chr2 + 204733604 204733630
RUNX1-1	TTTNTGTCTCTCTGCCCCCTCCCCACT	chr21 + 37371826 37371852
RUNX1-2	TTTNGCTCCGAAGGTAAAAGAAATCAT	chr21 - 36421242 36421268
DNMT1-3	TTTNCTGATGGTCCATGTCTGTTACTC	chr19 - 10244443 10244469
DNMT1-7	TTTNGCTCAGCAGGCACCTGCCTCAGC	chr19 - 10133918 10133944
EMX1	TTTNTCCTCCGGTTCTGGAACCACACC	chr2 - 73160921 73160947
IL1RN-1	TTTNCAGGAGGGTGACTCAGGCTAGCA	chr2 - 113875317 113875343
FANCF	TTTNTCCGTGTTCCTTGACTCTGGGCA	chr11 + 22647490 22647516
DYRK1A	TTTNGAAGCACATCAAGGACATTCTAA	chr21 + 38860226 38860252
KLF4	TTTNGTTTAAACACACCGGGTTAATAA	chr9 - 110247058 110247084
NPY1R	TTTNAAGCCTCGGGAAACTGCCCTGCC	chr4 - 164254002 164254028
CHN1	TTTNAGCTAAAACGCACACCGGCCCCG	chr2 - 175870275 175870301
SNX13-1	TTTNTCCTCTCCCCAGGGCGGGCCAAC	chr7 - 17980383 17980409
BRAF	TTTNGTCTAGCTACAGTGAAATCTCGA	chr7 - 140453126 140453152
mGreen-crRNA1	TTTCCAGGCCGCTATGGTGGATGGCAG	targeted mNeonGreen
mGreen-crRNA2	TTTCTTGCTTCTGCACCAGTCGGCGG	targeted mNeonGreen
mGreen-crRNA3	TTTGGCGAAGGTGTAGGTGGTCCGGGC	targeted mNeonGreen
mGreen-crRNA4	TTTGAAGTTCAGTTCTGTCTTGGAGTG	targeted mNeonGreen
IL1RN	TTTNCAGGAGGGTGACTCAGGCTAGCA	chr2 - 113875317 113875343
MYOD	TTTNCCGCGGATACAGCAGTCGGGTGT	chr11 + 17740805 17740831
HBG	TTTN CCTTGTCAAGGCTATTGGTCAAG	chr11 + 5271181 5271207
qPCR-IL1RN-F	GGAATCCATGGAGGGAAGAT	qPCR primer
qPCR-IL1RN-R	TGTTCTCGCTCAGGTCAGTG	qPCR primer
qPCR-MYOD-F	CCGACGGCATGATGGACTAC	qPCR primer
qPCR-MYOD-R	AGGCAGTCTAGGCTCGACAC	qPCR primer
qPCR-HBG-F	GCTGAGTGAACTGCACTGTGA	qPCR primer
qPCR-HBG-R	GAATTCTTTGCCGAAATGGA	qPCR primer
qPCR-GAPDH-F	AGAAGGCTGGGGCTCATTTG	qPCR primer
qPCR-GAPDH-R	AGGGGCCATCCACAGTCTTC	qPCR primer

Table S2. Deep-seq primers for this study

Name	Sequence(5'-3')
FANCF-DS-F	cgCTCTTTCCCTACACGACGCTCTTCCGATCTIndex1ACTGATTGGAACATCCGCGA
FANCF-DS-R	ctACTGGAGTTCAGACGTGTGCTCTTCCGATCTIndex2ATGACTGGCATCATCTCGCA
DYRK1A-DS-F	cgCTCTTTCCCTACACGACGCTCTTCCGATCT Index1TCAGCACAGAGCCATAGTCAC
DYRK1A-DS-R	tACTGGAGTTCAGACGTGTGCTCTTCCGATCT Index2AAGAGTGAGGAGAAGGCAGGA
