# Supplementary material for: Profiling the impact of the promoters on CRISPR-Cas12a system in human cells
Source: Cell Mol Biol Lett. 2023 May 17;28:41. doi: 10.1186/s11658-023-00454-9 (PMC10190037; doi:10.1186/s11658-023-00454-9)
Supplement: Supplementary file 2 — Additional file 2: Figure S1. Detection of the transfection efficiency by FACS. Figure S2. Detection of the editing specificity by Tag-seq. Figure S3. The specificity of the CRISPR-Cas12a systems using different promoters in HEK293T cells. Figure S4. The multiplex-editing specificity of the CRISPR-Cas12a systems with different promoters in HEK293T cells. Figure S5. The activation level of the Cas12a-based CRISPRa systems using different promoters in HEK293T cells. Figure S6. Base editing activity of the CRISPR-Cas12a systems with different promoters. [file 11658_2023_454_MOESM2_ESM.pdf]

Additional file 2

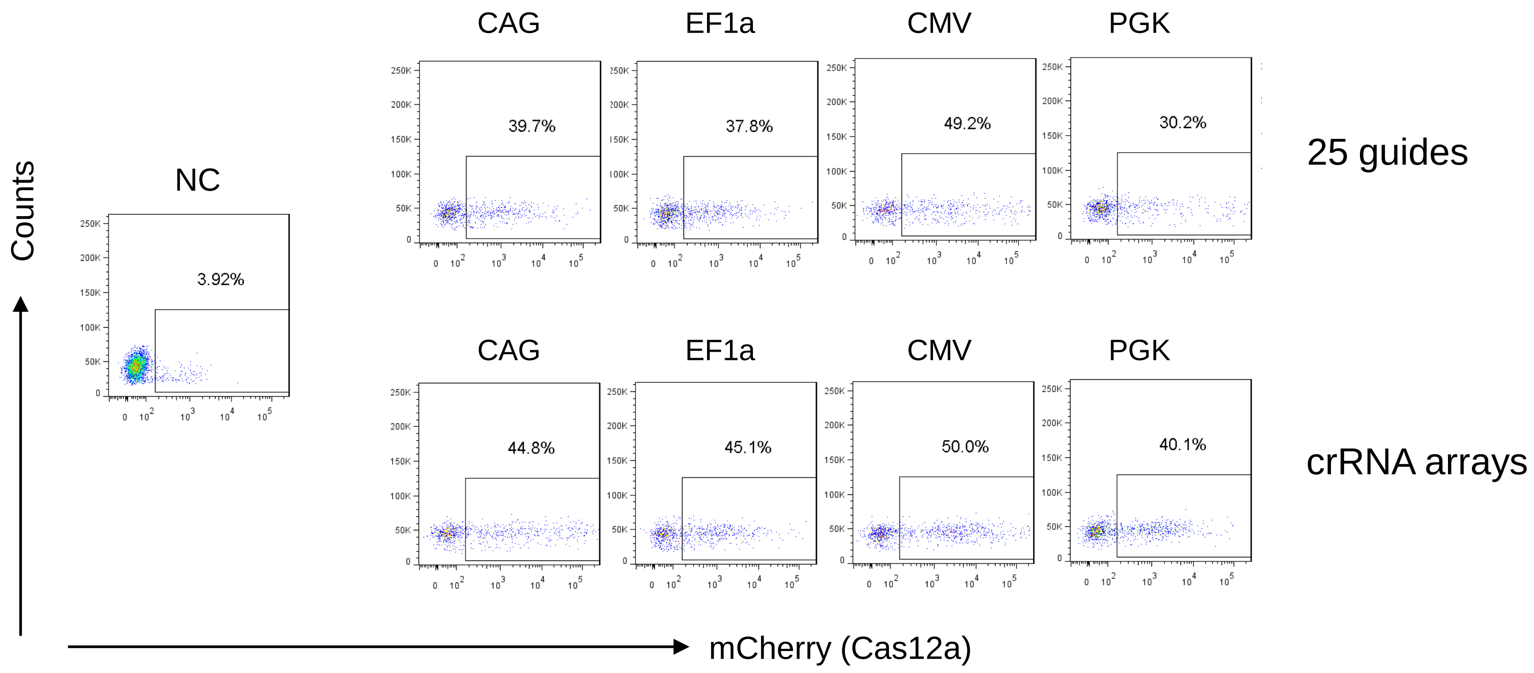

Figure S1. Detection of the transfection efficiency by FACS. The transfection were administrated by PEI-based method in 12-well plates with the plasmids expression of Cas-protein (fusing a P2A mCherry reporter, 600 ng) and the pooled crRNAs (up, 25 guides, total 600 ng) or the crRNA array (down, 6 sites, 600 ng), and an Tag-oligo DNA (10uM) in MCF7 cells, and the transfection efficiency was determined by FACS with the mCherry reporter.

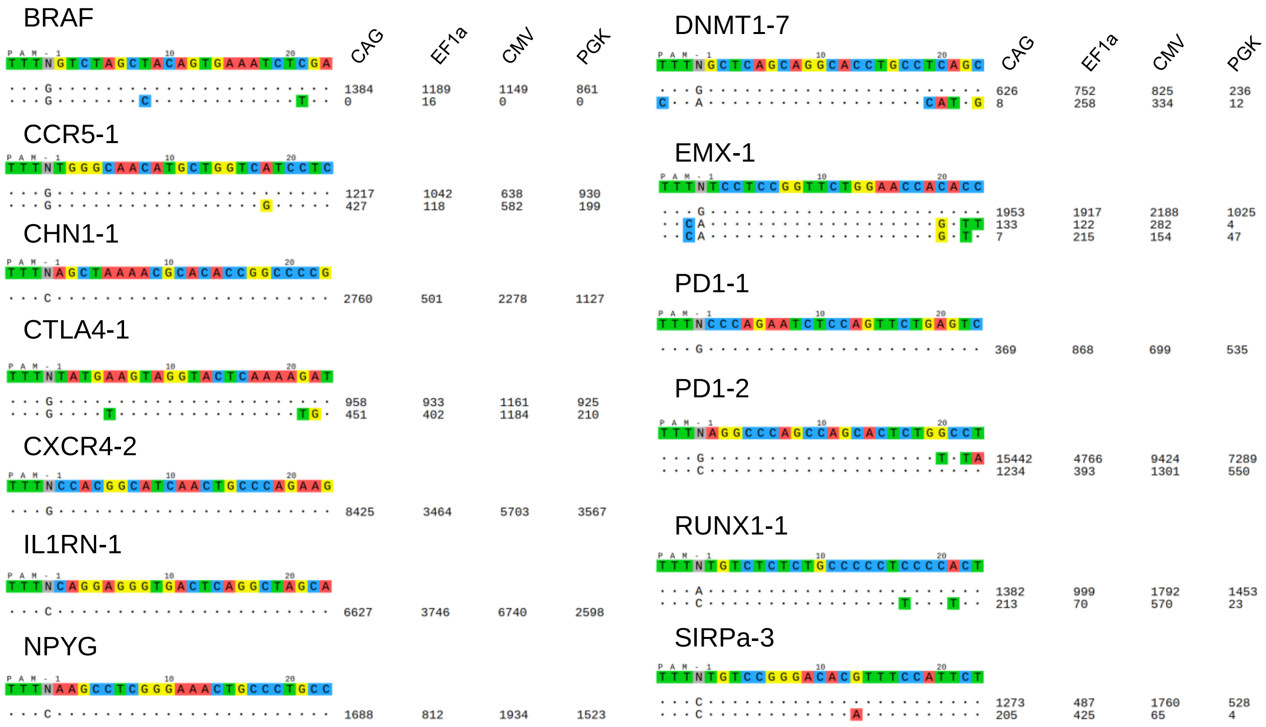

Figure S2. Detection of the editing specificity by Tag-seq. MCF7 cells were co-transfected with the plasmids expressing AsCas12a-HF driven by different promoters, a pooled twenty-five crRNAs, and the donor Tag sequence. Genomic DNA was harvested after three days post-transfection for libraries construction and Tag-seq analysis. Read counts represented a measure of cleavage frequency at a given site, mismatched positions showing the off-targets were highlighted in color within the spacer or PAM. (also see Fig. 2b)

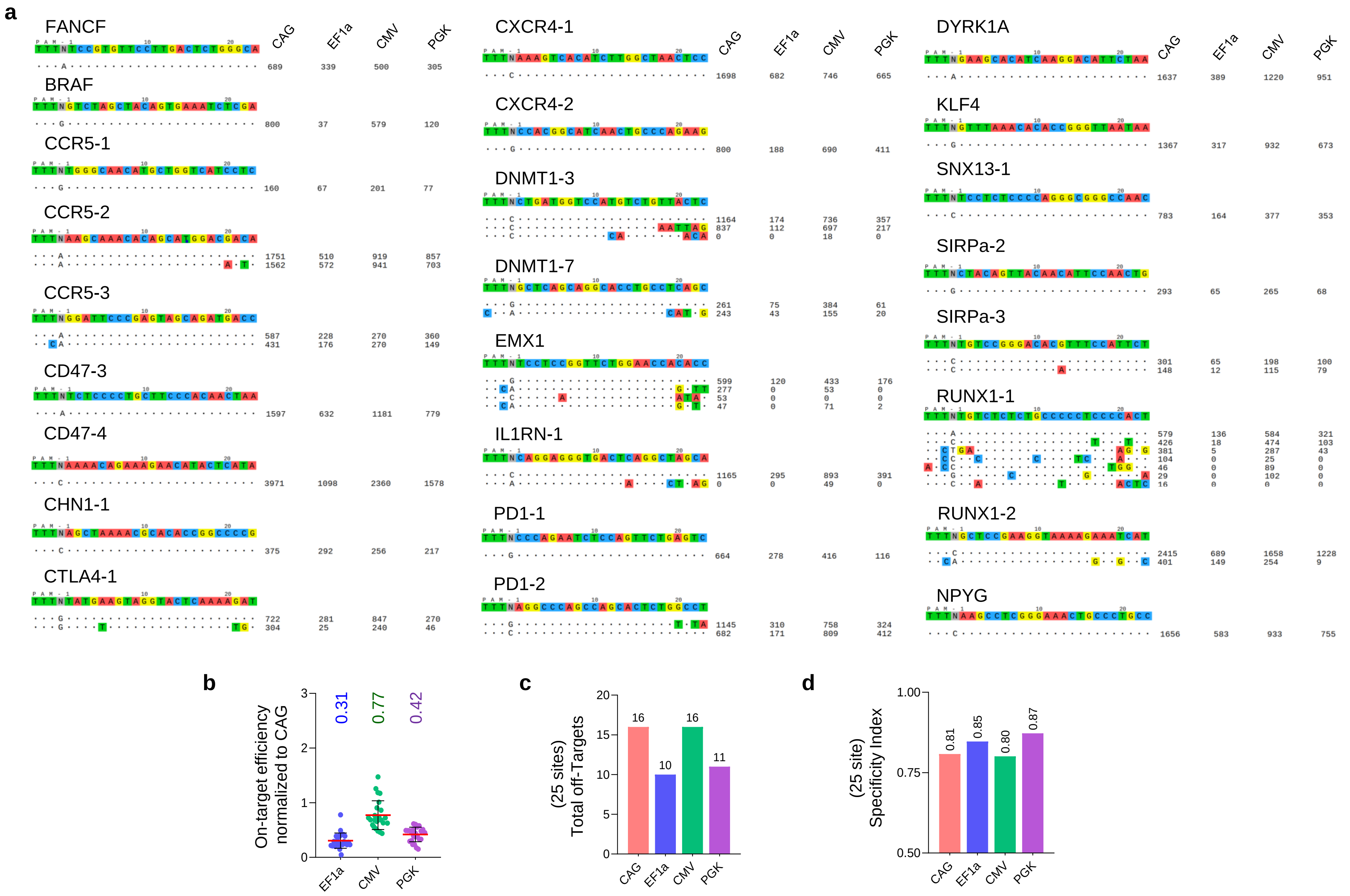

Figure S3. The specificity of the CRISPR-Cas12a systems using different promoters in HEK293T cells

Figure S3. The specificity of the CRISPR-Cas12a systems using different promoters in HEK293T cells. a Detection of the editing specificity by Tag-seq in HEK293T cells. HEK293T cells were co-transfected with the plasmids expressing AsCas12a-HF driven by different promoters, a pooled twenty-five crRNAs, and the donor Tag sequence. Read counts represented a measure of cleavage frequency at a given site, mismatched positions showing the off-targets were highlighted in color within the spacer or PAM. **b** Normalization of on-target activity of the various CRISPR-Cas12a systems to the CRISPR-Cas12a driven by the CAG promoter, value=(other systems on-target reads)/(CRISPR-Cas12a with CAG promoter). **c** Total number of off-target sites detected with the twenty-five crRNAs. **d** Specificity Index assessment (value was calculated by the ratio of total on-target reads to the on-target reads plus the off-target reads within the twenty-five sites).

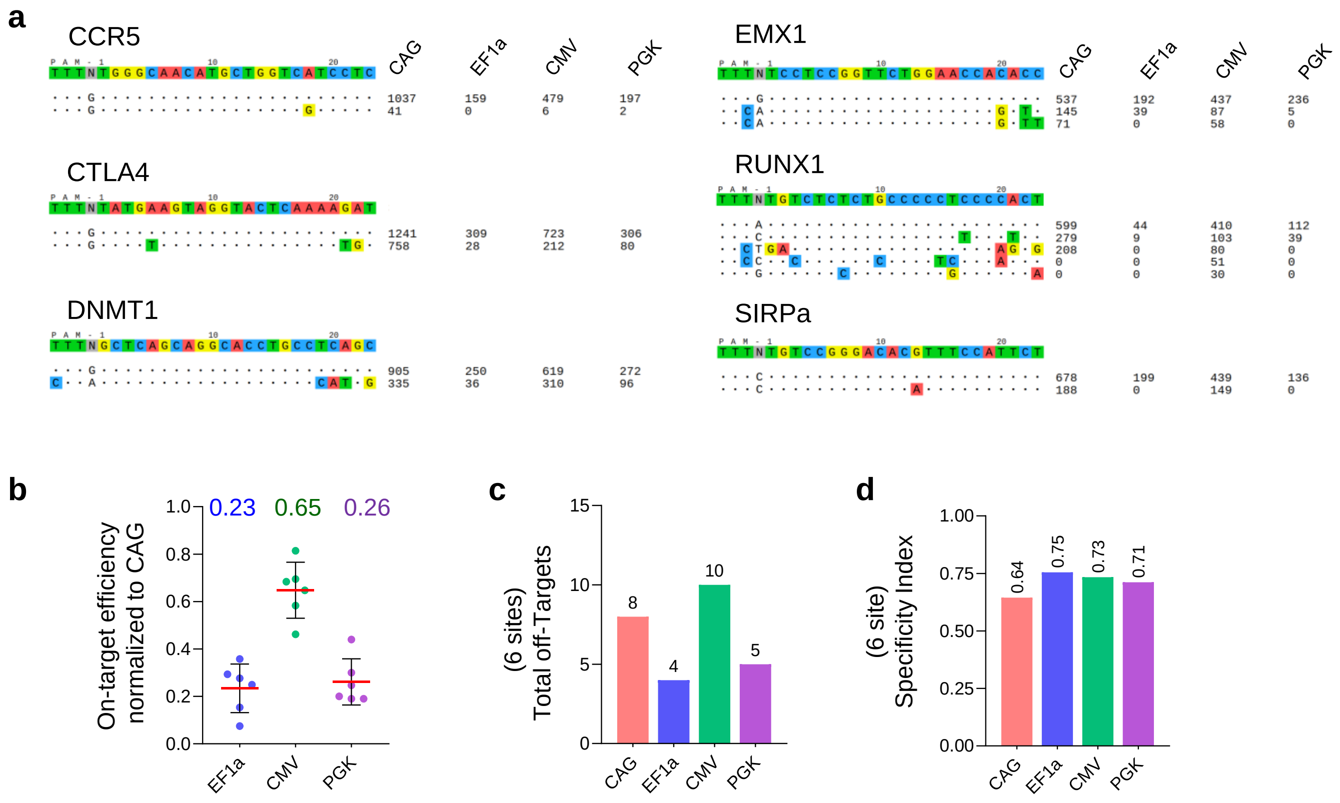

Figure S4. The multiplex-editing specificity of the CRISPR-Cas12a systems with different promoters in HEK293T cells

Figure S4. The multiplex-editing specificity of the CRISPR-Cas12a systems with different promoters in HEK293T cells. a Detection of the multiplex-editing specificity by Tag-seq in HEK293T cells. HEK293T cells were co-transfected with the plasmids expressing AsCas12a-HF driven by different promoters, a crRNA array targeting six sites, and the donor Tag sequence. Read counts represented a measure of cleavage frequency at a given site, mismatched positions showing the off-targets were highlighted in color within the spacer or PAM. **b** Normalization of on-target activity of the various CRISPR-Cas12a systems to the CRISPR-Cas12a driven by the CAG promoter, value=(other systems on-target reads)/(CRISPR-Cas12a with CAG promoter). **c** Total number of off-target sites detected with the six sites. **d** Specificity Index assessment (value was calculated by the ratio of total on-target reads to the on-target reads plus the off-target reads within the six sites).

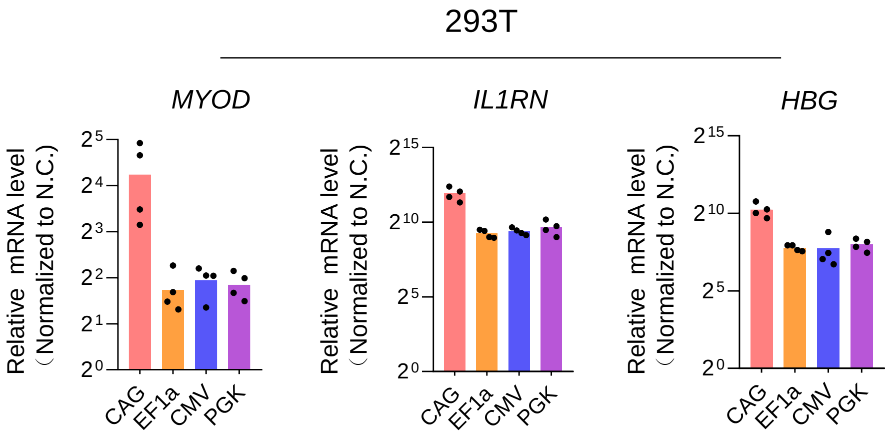

Figure S5. The activation level of the Cas12a-based CRISPRa systems using different promoters in HEK293T cells. a-c qPCR analysis of the transcriptional activation among the CRISPR-Cas12a systems with different promoters guided by a single crRNA targeting each promoter region of *MYOD* (a), *IL1RN* (b) and *HBG* (c) in human HEK293T cells. Mean values are presented with SEM, n=4 independent experiments.

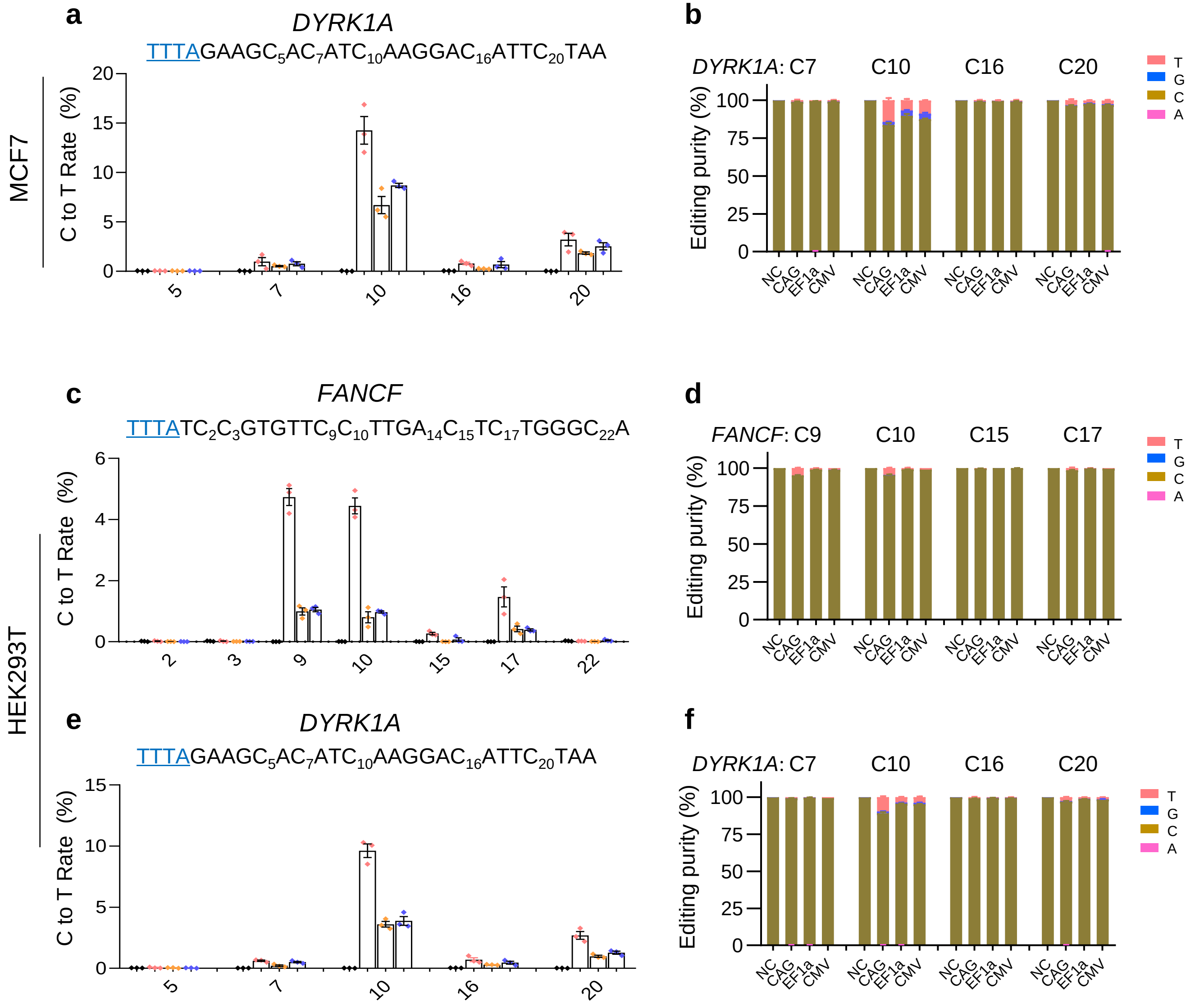

Figure S6. Base editing activity of the CRISPR-Cas12a systems with different promoters

Figure S6. Base editing activity of the CRISPR-Cas12a systems with different promoters. a Deep-seq revealed the cytosine to thymine (C-to-T) editing at *DYRK1A*-Site in MCF7 cells. **b** Analysis of the editing purity at *DYRK1A*-Site. The fraction was plotted by calculated each nucleotide reads within total reads at this site. **c** Deep-seq revealed the cytosine to thymine (C-to-T) editing at *FANCF*-Site in HEK293T cells. **d** Analysis of the editing purity at *FANCF*-Site. **e** Deep-seq revealed the cytosine to thymine (C-to-T) editing at *DYRK1A*-Site in HEK293T cells. **f** Analysis of the editing purity at *DYRK1A*-Site. For all the figures, mean values are presented with SEM, n=3 independent experiments. NC, cells transfected with a non-targeted crRNA.
